# Impact of in ovo feeding of grape pomace extract on the growth performance, antioxidant status, and immune response of hatched broilers

**DOI:** 10.1016/j.psj.2024.103914

**Published:** 2024-05-28

**Authors:** Mahmoud Madkour, Sayed A. Abdel-Fattah, Sami I. Ali, Nematallah G.M. Ali, Mohamed Shourrap, Mohamed Hosny, Ahmed A. Elolimy

**Affiliations:** ⁎ Animal Production Department, National Research Centre, Dokki, 12622, Giza, Egypt; † Poultry Production Department, Faculty of Agriculture, Ain Shams University, Shoubra El-Kheima, 11241, Cairo, Egypt; ‡ Plant Biochemistry Department, National Research Centre, Dokki, 12622, Giza, Egypt; § Department of Animal Production, Faculty of Agriculture, Al-Azhar University, Assiut 71524, Egypt; # Department of Integrative Agriculture, College of Agriculture and Veterinary Medicine, United Arab Emirates University, Al Ain, Abu Dhabi, United Arab Emirates

**Keywords:** in ovo feeding, grape pomace extract, growth performance, antioxidant status, broiler chicken

## Abstract

Delivering natural antioxidants via in ovo feeding holds promise for enhancing the antioxidant status and performance of chickens. Therefore, The objective of this study was to evaluate the impacts of in ovo feeding during early embryonic development using grape pomace extract as a natural antioxidant on hatchability, productive performance, immune response, and antioxidant status in broilers. A total of 900 fertile broiler eggs from the Arbor Acres strain were utilized. Each egg was individually weighed, with egg weights ranging from 61.88 ± 3 g. On the 17.5th d of incubation (**DOI**), the fertile eggs were divided into 6 groups. The first treatment group was untreated and designated as the control (**C**). The second group was the sham group (**Sh**), receiving a simulated injection. The third group, designated as the vehicle group (**V**), was injected with 100 µl of dimethyl sulfoxide (**DMSO**). The fourth group received an injection of 100 µL of grape pomace dissolved in DMSO at a concentration of 2 mg (T2). Similarly, the fifth and sixth groups were injected with 100 µL of grape pomace dissolved in DMSO at concentrations of 4 mg and 6 mg, (T4), (T6) respectively. Subsequently, all groups were raised under uniform conditions in terms of management, environment, and nutrition till 5 wk of age. The grape pomace extract (**GPE**), obtained is rich in total phenolic content (16.07 mg/g), total flavonoid content (7.42 mg/g), and total anthocyanin (8.37 mg/g). Grape pomace extract has exhibited significant antioxidant properties as evidenced by its effectiveness in DPPH scavenging and reducing power assays. Significant improvements in body weight at hatch were observed with in ovo feeding of grape pomace extract, particularly at the 4 mg level, surpassing the effectiveness of the 2 mg and 6 mg grape pomace levels, and this enhancement in body weight continued until the age of 5 wk. GPE injection also led to a significant reduction in cholesterol levels, with the lowest levels recorded for the T4 group. Plasma total Antioxidant Capacity (**TAC**) levels were significantly elevated in groups treated with T4, T6, and T2 compared to the control group. Conversely, the control group showed a significant increase (*P* < 0.01) in plasma malondialdehyde (MDA) levels. The immune response of hatched chicks from grape pomace extract-injected groups, especially the T4 group, exhibited improvement through increased IgM and IgG. These findings demonstrate that in ovo feeding of GPE, particularly at a dosage of 4 mg, enhances growth performance, immune response, and antioxidant status in hatched chicks. Thus, administering natural antioxidants, such as grape pomace extract, to developing broiler embryos via in ovo feeding could serve as a valuable strategy for enhancing the subsequent post-hatch productive performance, as well as bolstering the antioxidant and immunological status of broiler chicks.

## INTRODUCTION

In ovo inoculation, recognized as a technology, has been extensively employed in embryo vaccination procedures during artificial incubation. Numerous studies have indicated that the introduction of external nutrients to the amnion of avian embryos in the advanced stages of development could accelerate the development of the growing embryo both before and after hatching (Zhang et al., 2018; Das et al., 2021; Abd El- Azeem et al., 2024). The chicken embryo as a kind of precocial species, there is a rapid increase in oxygen consumption and metabolic rate during and shortly after hatching. This surge is necessary to meet the demands of endothermy and locomotion, facilitated by the transition from chorioallantoic to pulmonary respiration (Hohtola, 2002). To cope with this sudden exposure to oxidative stress, it would be beneficial to have a well-established and effective antioxidant system. It is noteworthy that the tissues of chick embryos contain a substantial amount of highly polyunsaturated fatty acids in the lipid fraction (Surai et al., 2016), necessitating antioxidant defense mechanisms (Abd El-Azeem et al., 2014). The early post-hatching period and last stage of embryonic development are pivotal stages in the lifecycle of broiler chickens. moreover, a detrimental effect on the immune capability of hatched chicks is noticeable due to delayed access to food, rendering them more susceptible to diseases and leading to increased mortality rates (Abd El- Azeem et al., 2024). Delivering natural antioxidants via in ovo feeding holds promise for enhancing both the antioxidant status and performance of chickens. (Akosile et al., 2023). Lately, there has been an increasing inclination toward utilizing phytochemicals (such as polyphenols, isoflavones, and carotenoids) obtained from natural sources due to their potential antioxidant activities (Alagawany et al., 2022; Wani et al., 2022; Madkour et al., 2024). Grape pomace represents a valuable reservoir of polyphenols, comprising around 30 to 40% depending on the grape variety and processing methods. The polyphenols found in grape pomace predominantly include catechins, flavonols, anthocyanins, and stilbenes, notably resveratrol, along with various phenolic acids derived from benzoic and cinnamic acids (Ky and Teissedre, 2015) . Polyphenolic compounds offer numerous health benefits, primarily due to their strong antioxidant properties. These bioactive polyphenolics act as reducing agents in various biological systems by supplying hydrogen, reducing singlet oxygen, chelating substances, and scavenging free radicals(Zhang and Tsao, 2016). These results motivated us to assess the impact of in ovo feeding during early embryonic development using grape pomace extract as a natural antioxidant. We examined its effects on hatchability, body weight at hatch, antioxidant status, and immune response in hatched chicks. Furthermore, we investigated how these factors influenced the overall productive performance of broilers up to the market age.

## MATERIAL AND METHODS

The animal study protocol, with ethical approval code (5-2023-9), was granted approval by the Ethics Committee of Ain Shams University, Agriculture sector committee.

### Preparation of Extract

The pulverized grape pomace, including fruit skin and seeds, and stalks of grapevine, (800 g) was soaked with methanol 80% in water three times. The ratio of grape pomace powder to solvent was 1:5. The supernatants obtained after centrifugation were pooled, concentrated under vacuum at 45°C, and subsequently lyophilized to produce an 80% methanol crude extract of grape pomace (**GPE**). The lyophilized GPE was then dissolved in DMSO for further analysis

### Phytochemical Analysis

#### Assessment of Total Phenolic Content

The Folin-Ciocalteu assay was employed to assess the total phenolic content (**TPC**) in the GPE, following the procedure outlined by (Mohamed et al., 2016). The results were quantified as milligrams of gallic acid equivalents (**GAE**) per gram of grape pomace dry weight.

#### Total Flavonoid Content

We applied the aluminum chloride method to evaluate the total flavonoid content (**TFC**) in the GPE, employing the procedure outlined by (Mohamed et al., 2015). The results were presented as milligrams of quercetin equivalents (**QE**) per gram of grape pomace dry weight

#### Total Anthocyanin Content

The total anthocyanin content (**TAC**) of methanol 80% crude extract of grape pomace was calculated using E value (98.2, mg/100g) of Cyanidin-3-glucoside chloride followed the cranberry anthocyanin quantification developed by (Fuleki and Francis, 1968). The total anthocyanin content in the sample was quantified and expressed as milligrams of Cyanidin-3-glucoside chloride equivalents (C3G) per gram of dry weight.

#### HPLC Analysis of Phenolic Ingredients

We vortexed 10 mg of GPE in 2 mL methanol for 15 min., then filtrated it using A 0.2 μm Millipore membrane filter. We injected the filtrate (5 μL) into an HPLC (Agilent Technologies 1,260 series, Germany) utilizing its auto-sampling injector. We isolated phenolic compounds at a constant temperature of 40 ± 2°C and 0.9 mL/min flow rate using a 4.6 mm x 250 mm Eclipse C-18 column with a particle size of 5 μm. The mobile phase consisted of a blend of water (A) and acetonitrile with 0.05% trifluoroacetic acid (B), with a linear gradient as follows: 0 min (82% A), 0 to 5 min (80% A), 5 to 8 min (60% A), 8 to 12 min (60% A), 12 to 15 min (82% A), and 15 to 20 min (82% A). The DAD detector was set to 280 nm (Gaafar Alaa et al., 2018). The concentration of each specific phenolic component (µg/g of extract) is calculated by comparing its relative peak area with the reference.

### Antioxidant Capacity

#### DPPH• Radical Scavenging Assay

We evaluated the DPPH• radical scavenging activity of GPE at concentrations of 25, 50, 75, and 100 μg/mL using the method outlined by (Gaafar et al., 2020). Butyl hydroxytoluene (**BHT**) served as the positive control. The results were presented as the percentage of DPPH• scavenging using the formula: DPPH scavenging (%) = (Ac - As) / Ac × 100, where Ac represents the control reading and As represents the sample reading.

#### Reducing Power Assay

We evaluated the reducing power efficacy of GPE at concentrations of 25, 50, 75, and 100 μg/mL using the method outlined by (Ali et al., 2018). Butyl hydroxytoluene (**BHT**) was employed as the positive control. The results were quantified as absorbance at 700 nm.

#### Experimental Procedure

A total number of 900 fertile broiler eggs from (**Arbor Acres strain**) used in this experiment, their weights ranged from (61.88 ± 3 g). The incubation conditions were arranged as implemented by (Hemida et al., 2023; Abdel-Fattah et al., 2024). On the 7th and 17th d of incubation (**DOI**), the eggs were manually candled to remove infertile eggs and early dead embryos.

$$\text{Fertility}\%=\frac{\text{Total}\;\text{eggs}-\;\text{clear}\;\text{eggs}\;}{\text{Total}\;\text{eggs}\;}X\;100$$

#### Experimental Treatments

live embryos were candled to determine the injection site. Then, eggs were divided into six groups of 130 eggs each and treated as follows: 1st group was the control while 2nd group Sham, 3rd vehicle group was injected with dimethyl sulfoxide (DMSO -Sigma-Aldrich co.). The 4th, 5th^,^ and 6th groups were injected (2, 4, and 6 mg/egg) with grape pomace extract (**GPE**). the experimental treatments as follow.

#### Injection Procedure

At the 17.5th DOI, eggs were sterilized with 70% ethyl alcohol. All injections were administered using a 1ml insulin syringe. the syringe contents were administered into the amnion as reported by (Abd El-Azeem et al., 2014).

Experimental Treatments

**Table:** 

Symbol	Description
C	Eggs were un-injected and served as (negative control).
Sh	Eggs were simulated injection without any material.
V	Vehicle dimethyl sulfoxide (DMSO) was injected with 100 μl (positive control).
T 2	Grape Pomace Extract (GPE) was injected with 100 μl (of 2 mg GP dissolved in DMSO)
T 4	Grape Pomace Extract (GPE) was injected with 100 μl (of 4 mg GP dissolved in DMSO)
T 6	Grape Pomace Extract (GPE) was injected with 100 μl (of 6 mg GP dissolved in DMSO)

Each hatched chick was weighed individually, and their weights (in grams) were recorded, and hatchability percentages were calculated.

Hatchability%=(Totalhatchedchicks)/(Totalfertileeggs)×100

#### Housing and Diets

The hatched chicks were allocated into six treatment groups, with each treatment group comprising 6 replicate pens. The floor pens measured approximately 1.22 m × 2.44 m and were furnished with fresh pine shavings as the litter material. During the first week, the ambient temperature was maintained at 33°C, followed by 28°C during the second week, and subsequently 25°C. The house was ventilated artificially, and the relative humidity was maintained at 50 ± 5% throughout the experiment. Chicks were provided with unrestricted access to a commercial basal diet formulated to meet the recommended requirements. Fresh water was continuously available through automatic nipple drinkers.

### Data Collection

#### Growth Performance

The weekly assessment of feed intake and live body weights was conducted on a replicate basis throughout the experiment using a precise scale, with daily monitoring of mortality. Feed conversion ratio and weight gain were calculated, and adjustments were made for mortality. Feed conversion was computed as the ratio of feed consumed to weight gain.

#### Plasma Measurements

Upon hatching, twelve birds from each treatment were randomly selected and slaughtered. Subsequently, two blood samples were combined into one sample, resulting in 6 samples from each group. Plasma stored at (-20°C) until the time of analysis. Plasma total protein (g/dL), albumin (g/dL) based on a colorimetric method, globulin was calculated by subtracting albumin from total plasma proteins for each sample and then A/G ratio was calculated. Cholesterol (mg/dL), triglycerides (mg/dL), HDL cholesterol (mg/dL) while LDL was calculated.

The activities of aspartate aminotransferase (**AST**) and alanine aminotransferase (**ALT**) were assessed colorimetrically using commercial kits (Diamond Diagnostics Co., Cairo, Egypt). Plasma thyroxin (**T4**) and triiodothyronine (**T3**) were quantified using ELISA kits obtained from Precheck Bio. Company. Subsequently, the T3/T4 ratio was calculated. Chicken plasma immunoglobulin IgM was quantified using the Chicken IgM ELISA Kit (Ab157692), while plasma IgG was assessed using the chicken IgG ELISA kit (MyBioSource, Cat No. MBS260043). The methodology employed a double-sandwich ELISA technique, as previously reported (Mallery et al., 2010). Antioxidant status was assayed by a colorimetric technique using commercial kits. Malondialdehyde (**MDA**),and total antioxidant capacity were calorimetrically determined using commercial enzyme Kits. Biodiagnostic Company (Dokki, Giza, Egypt).

#### Statistical Analysis

The data collected in this study were statistically analyzed using the GLM procedure of the SAS program (SAS, 2004). One-way analysis of variance was applied to all the data to compare the effect of different test groups according to the control group .

## RESULTS

### Phytochemical Analysis

**Figure 1:**
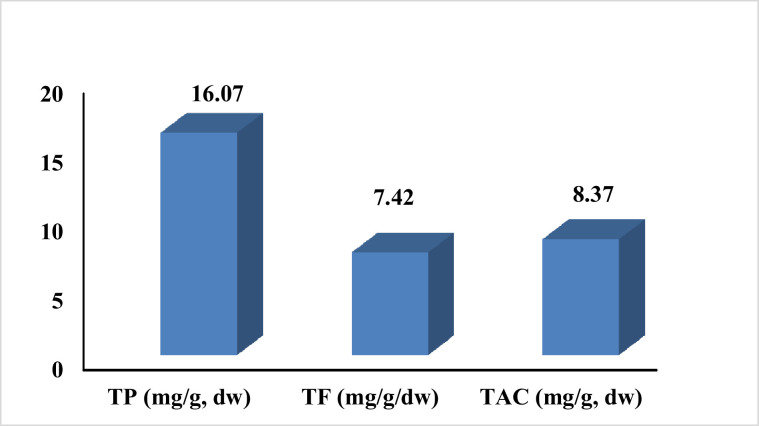
Total phenolic content (TPC, mg/g dry weight), total flavonoids content (TFC, mg/g dry weight), and total anthocyanin content (TAC, mg/g dry weight) present in grape pomace extract (**GPE**).

**Figure 2:**
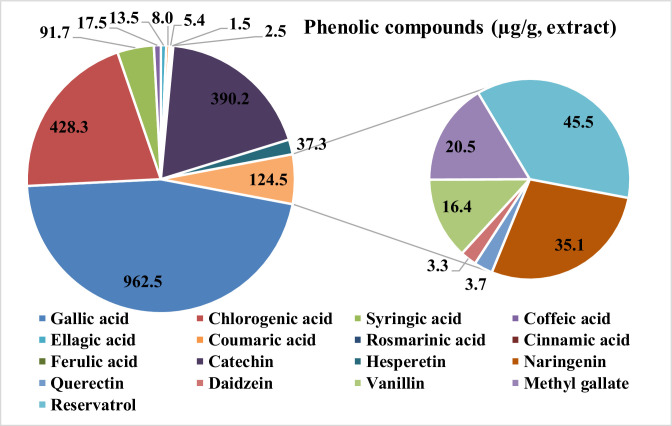
HPLC analysis of polyphenolic components present in grape pomace extract (**GPE**).

The phytochemical analysis displayed that grape pomace extract (**GPE**) is rich in total phenolic content (16.07 mg/g dry weight), total flavonoid content (7.42 mg/g dry weight), and total anthocyanin (8.37 mg/g dry weight), as presented in Figure 1. Figure 2 displays the results of the HPLC analysis of the polyphenolic components in GPE, which identified and quantified seventeen phenolic compounds. The following nine phenolic acids were quantified in GPE in descending order: gallic acid, chlorogenic acid, syringic acid, coffeic acid, ellagic acid, coumaric acid, rosmarinic acid, ferulic acid, and cinnamic acid. The following five flavonoids were quantified in GPE in descending order: catechin, hesperetin, naringenin, quercetin, and daidzein. In addition to phenolic acids and flavonoids, GPE contains resveratrol (45.5 µg/g extract) as presented in Figure 2.

### Antioxidant Activity

**Figure 3:**
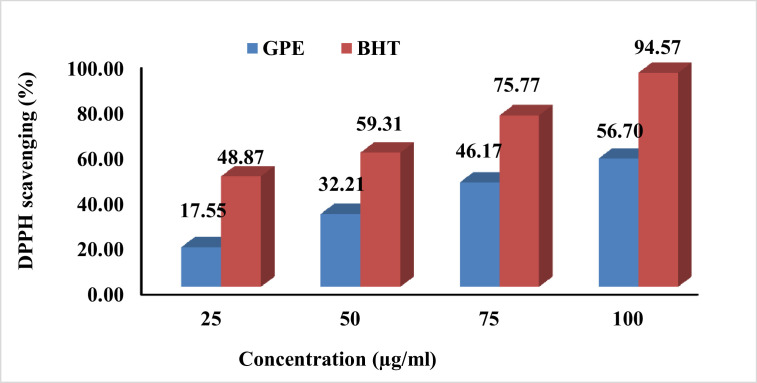
DPPH scavenging effect (%) of grape pomace extract (**GPE**).

**Figure 4:**
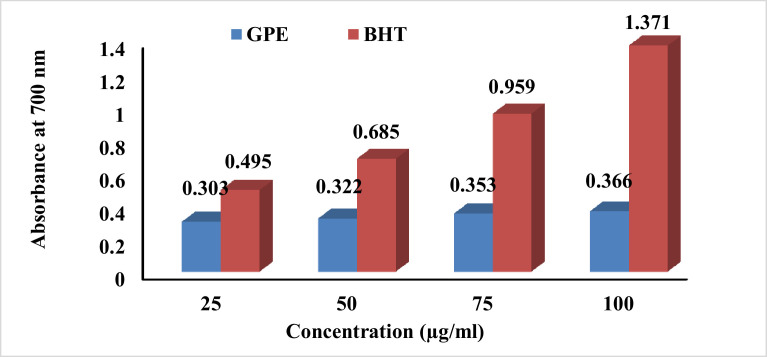
Reducing power effect (absorbance at 700 nm) of grape pomace extract (**GPE**).

The results of the antioxidant assays, DPPH and reducing power, indicate that (GPE) has potent antioxidant effects. The antioxidant effect of GPE is dependent on the concentration of the extract, as it increases with higher concentrations. For instance, at a concentration of 100 µg/mL, GPE showed a potent DPPH scavenging effect of 56.7% (Figure 3) and a reducing power effect with an absorbance reading of 0.366 at 700 nm (Figure 4). However, these antioxidant effects of GPE were still lower than those of the positive control, BHT.

### Impact of In Ovo Injection of Grape Pomace Extract on the Percentage of Hatchability

The hatchability percentages are displayed in Table 1. The hatchability percentage was significantly impacted by the treatments. Specifically, when considering the in ovo treatments as the primary factor, the data indicates that the T6 treatment exhibited the lowest hatchability percentage (89.82%), while T4 showed the highest value (91.8%) compared to other treatments.

**Table 1:** Effect of *in ovo* injection of grape pomace extract on hatchability percentage.

Experimental groups						
Traits	Control	Sham	V	T2	T4	T6
**Hatchability (%)**	92.8	91.1	90.8	91.83	91.2	89.82

V, vehicle control (sterilized dimethyl sulfoxide (DMSO).T2 =2mg Grape Pomace dissolved in DMSO, T4=4mg Grape Pomace dissolved in DMSO, T6 =6mg Grape Pomace dissolved in DMSO.

### Impact of In Ovo Injection of Grape Pomace Extract on Productive Performance

The data in Table 2 demonstrates a significant enhancement in body weight at hatch through in ovo injection with GPE compared to the control group. The 4 mg level exhibited greater effectiveness than the 2 mg and 6mg grape pomace levels. Furthermore, this improvement in body weight persisted until 5 wk of age.

**Table 2:** Effect of *in ovo* injection of grape pomace extract on live body weight (g) and body weight gain (g/ bird) at 5 wk of age**.**

			Experimental groups				SEM	Prob.	Sig.
			Grape pomace extract						
Age(wk)	Control	Sham	V	T2	T4	T6			
**Live body weight (LBW(g))**									
**At hatch**	44.65^c^	44.89^bc^	45.39^bc^	46.31^bc^	46.76^a^	46.45^ab^	1.79	0.04	**
**5 wk**	1766.0^c^	1827.51^bc^	1839.69^b^	1860.7^ab^	1915.91^a^	1888.89^a^	13.17	0.008	**
**Body weight gain (BWG(g/bird))**									
**0-5**	1726^c^	1786.13^b^	1798.1^ab^	1819.2^ab^	1875.13^a^	1847.27^a^	2.828	0.001	**
**Feed consumption (FC(g/bird))**									
									
**0-5**	2969.7	2904.3	2976.4	2908.85	2820.2	2843.75	6.23	0.148	NS
**Feed conversion ratio (FCR)**									
**0-5**	1.72^a^	1.63^ab^	1.66^b^	1.60^b^	1.50^b^	1.54^b^	0.021	0.019	*

SEM, standard error of means**. a, b, c…** means in the same Column within each classification bearing different letters are significantly different. sig. =Significance. NS=non-significant. V=Vehicle control (sterilized dimethyl sulfoxide (DMSO).T2 =2mg Grape Pomace dissolved in DMSO, T4=4mg Grape Pomace dissolved in DMSO, T6 =6mg Grape Pomace dissolved in DMSO.

The injection of grape pomace extract significantly improved LBW (*P* < 0.01) across all experimental periods compared to the control group. T4 consistently exhibited the highest LBW values throughout the experimental phases. Additionally, the final LBW values were notably higher for the T4, T6, and T2 groups, showing an increased ratio compared to the control group by (8.5, 7, and 5.4%, respectively). The differences between the vehicle, sham group, and control group were not significant during the experimental periods.

A comparable pattern was noted in the values of BWG, indicating a significant (*P* < 0.01) increase among all treated groups compared to the control group. As anticipated, groups T4, T6, and T2 demonstrated the highest overall BWG values, with an increment ratio of (8.8, 7.1, and 5.5%, respectively) In comparison to the control group.

As indicated in Table 2, the feed consumption (**FC**) of broiler chicks did not show a significant (*P* > 0.05) change due to in ovo feeding with grape pomace extract. However, there were insignificant decreases in FC for broilers from the T4 and T2 in ovo injected groups, respectively. Nevertheless, a distinct pattern emerged in the feed conversion ratio (**FCR**) values, demonstrating a significant enhancement (*P* < 0.05) in all injected groups over the entire duration (0–5 wk of age). The groups achieved the most favorable FCR values overall (T4, T6, T2, and sham) at (1.50, 1.54, 1.60, and 1.63), respectively.

### Influence of In Ovo Injection of Grape Pomace Extract on Specific Blood Biochemistry Parameters at Hatch

The findings presented in Table 3 indicate that in ovo injection with 4mg of grape pomace extract significantly increased the plasma total proteins (**PTP**) of hatchling chicks, suggesting a positive effect on protein metabolism. Additionally, at hatch, plasma albumin was significantly elevated in the T4 group of chicks, followed by T6 and T2, in comparison to the control group, implying a potential impact on liver activity due to grape pomace extract treatment. The plasma globulin of chicks also experienced a significant increase with grape pomace extract at hatch, suggesting an enhancement in immunity, with the highest value observed in the T4 treatment. Although the A/G ratio values were not significantly different from the control, numerically, various treatments recorded higher values than the control, particularly T4 and T6.

**Table 3:** Effect of *in ovo* injection of grape pomace extract on some plasma protein at hatch.

Experimental groups							Sig.	Prob.	SEM
Grape pomace extract									
T6	T4	T2	V	Sham	Control	**Traits**			
**Total protein(g/dL)**	2.99^c^	3.28^c^	3.53^bc^	4.49^b^	4**.**85^a^	4.68^ab^	0.109	0.0001	**
**Albumin(g/d)**	1.35^c^	1.50^c^	1.60^bc^	2.07^b^	2.12^a^	2.23^ab^	0.04	0.0001	**
**Globulin(g/d)**	1.64^d^	1.78^d^	1.93^c^	2.42^bc^	2.73^a^	2.45^b^	0.07	0.0001	**
**A/G ratio**	0.82	0.86	0.83	0.87	0.89	0.91	0.01	0.86	NS

SEM, standard error of means**. ^a, b,c^**… means in the same Column within each classification bearing different letters are significantly different. sig. =Significance. NS, non-significant, *= (*P* ≤ 0.05), **= (*P* ≤ 0.01). V=Vehicle control (sterilized dimethyl sulfoxide (DMSO).T2 =2mg Grape Pomace dissolved in DMSO, T4=4mg Grape Pomace dissolved in DMSO, T6 =6mg Grape Pomace dissolved in DMS.

Concerning the lipid profile (Table 4), the control group exhibited the highest levels of triglycerides and cholesterol, while the T4 group reported the lowest levels. The injection of grape pomace extract resulted in a significant reduction in cholesterol levels. Comparable patterns were noted for triglyceride values, with the lowest levels recorded for the T4 grape pomace extract group. Moreover, the significant differences in HDL levels among various treatments are evident (*P* ≤ 0.001), with T4 showing the highest concentration compared to other treatments and the control. While there were no significant differences between treatments for LDL concentrations, numerically, T4, T2, and T6 had the lowest concentrations compared to other treatments and the control.

**Table 4:** Effect of *in ovo* injection of grape pomace extract on lipids profile at hatch.

			Experimental groups				SEM	P≤0.01.	Sig.
			Grape pomace extract						
**Traits**	Control	Sham	V	T2	T4	T6			
Cholesterol (mg/dL)	142.77^a^	138.52^a^	132.0^b^	125.37^bc^	123.91^c^	127.6^c^	45.57	0.002	**
Tri-glycerides (mg/dL)	131.75^a^	130.67^a^	125.45^ab^	114.50^b^	112.20^c^	117.64^ab^	34.20	0.002	**
HDL-Cholesterol (mg/dL)	60.2 ^c^	62.4^c^	66.4^bc^	69.5^b^	77.7^a^	75.89^ab^	1.98	0.001	**
LDL- Cholesterol (mg/dL)	56.22	49. 99	40.51	32.97	23.77	28.18	2.48	0.08	NS

SEM, standard error of means**. ^a, b,c^**… means in the same Column within each classification bearing different letters are significantly different. sig. =Significance. NS=non-significant, *= (*P* ≤ 0.05), **= (*P* ≤ 0.01). V=Vehicle control (sterilized dimethyl sulfoxide (DMSO).T2 =2mg Grape Pomace dissolved in DMSO, T4=4mg Grape Pomace dissolved in DMSO, T6 =6mg Grape Pomace dissolved in DMS. HDL: high-density lipoprotein; LDL: low-density lipoprotein.

### Impact of In Ovo Injection of Grape Pomace Extract on Plasma Thyroid Gland Function Upon Hatching

The data presented in Table 5 indicates that the in ovo injection of grape pomace extract significantly elevated the plasma concentrations of thyroid gland hormones (T3 and T4) and the conversion ratio (T3/T4 ratio) in comparison to the control group. Notably, the control group had the lowest concentration, which was statistically significant (P ≤ 0.01) compared to all the experimental treatments.

**Table 5:** Effect of *in ovo* injection of grape pomace extract on plasma thyroid gland function at hatch.

			Experimental groups				SEM	Prob.	Sig.
			Grape pomace extract						
**Traits**	Control	Sham	V	T2	T4	T6			
**T_3_ (ng/mL)**	3.35^c^	3.78^b^	4.40^a^	4.49^a^	4.42^a^	3.78^b^	0.093	0.001	**
**T_4_ (ng/mL)**	13.53^c^	14.76^b^	16.24^a^	16.51^a^	15.92^a^	14.76^b^	0.223	0.001	**
**T_3_ / T_4_ratio**	0.25^b^	0.26^b^	0.27^a^	0.27^a^	0.28^a^	0.26^b^	0.005	0.05	*

SEM, standard error of means**. ^a, b,c…^** means in the same Column within each classification bearing different letters are significantly different. sig. =Significance. NS=non-significant, *= (*P* ≤ 0.05), **= (*P* ≤ 0.01). V=Vehicle control (sterilized dimethyl sulfoxide (DMSO).T2 =2mg Grape Pomace dissolved in DMSO, T4=4mg Grape Pomace dissolved in DMSO, T6 =6mg Grape Pomace dissolved in DMS. T4= thyroxine, T3= triiodothyronine.

### The Impact of In Ovo Injection of Grape Pomace Extract on Liver Function and the Antioxidant Status of Plasma During Hatching

The effect of in ovo injection of GPE on the activities of alanine aminotransferase (**ALT**) and aspartate aminotransferase (**AST**) of chicks at hatch was presented in Table 6. It is clear from these results that AST and ALT activity at hatch were significantly reduced in T4 and followed by T2 treatment groups compared to the control. In terms of antioxidant status, the Total Antioxidant Capacity (**TAC**) levels in plasma were significant elevated in groups treated with T4, T6, and T2 compared to the control group. Conversely, the levels of plasma Malondialdehyde (**MDA**) were significantly higher (*P* < 0.01) in the control and sham groups. Among the treated groups, the lowest MDA content was observed in T4 and T6 (0.13 nmol/ml), followed by group T2 (0.16 nmol/ml).

**Table 6:** Effect of *in ovo* injection of grape pomace extract on liver function and antioxidants status at hatch.

			Experimental groups				SEM	Prob.	Sig.
			Grape pomace extract						
**Traits**	Control	Sham	V	T2	T4	T6			
**AST (U/L)**	63.18^a^	57.63^ab^	48.10^b^	41.22^c^	43.66^c^	44.16^c^	0.093	0.001	**
**ALT (U/L)**	6.70^a^	6.33^a^	5.83^ab^	5.44^b^	4.90^c^	5.00^b^	0.223	0.001	**
**TAC (mM/L)**	0.43^c^	0.46^c^	0.55^b^	0.63^b^	0.77^a^	0.69^a^	0.04	0.05	*
**MDA (nmol/mL)**	0.20^a^	0.24^a^	0.16^b^	0.18^b^	0.13^c^	0.13^c^	0.0002	0.001	**

SEM, standard error of means**. ^a b,c^**… means in the same Column within each classification bearing different letters are significantly different. sig. =Significance. NS=non-significant, *= (*P* ≤ 0.05), **= (*P* ≤ 0.01). V=Vehicle control (sterilized dimethyl sulfoxide (DMSO).T2 =2mg Grape Pomace dissolved in DMSO, T4=4mg Grape Pomace dissolved in DMSO, T6 =6mg Grape Pomace dissolved in DMSO,TAC: AST: aspartate aminotransferase; ALT: alanine aminotransferase,Total antioxidant capacity, MDA: malondialdehyde.

### Impact of In Ovo Injection of Grape Pomace Extract on Plasma Immunoglobulin Levels (IgM, IgG, and total Ig) at Hatch

The impact of in ovo feeding of GPE on plasma immunoglobulin (IgM and IgG) levels of hatched chicks is depicted in Table 7. A significant increase (*p* ≤ 0.01) in plasma IgM and IgG was observed with higher grape pomace extract levels. Specifically, plasma IgG concentration showed an increase of 8.5% and 13.1% for the two grape pomace extract treatments (T4 and T6), respectively, in comparison to the control level.

**Table 7:** Effect of *in ovo* injection of grape pomace extract on plasma immunoglobulins (IgG, IgM and total Ig) at hatch.

			Experimental groups				SEM	Prob.	Sig.
			Grape pomace extract						
Traits	Control	Sham	V	T2	T4	T6			
**Ig M (mg/dL)**	281.89 ^c^	281.44^c^	284.67^c^	296.01^b^	318.57^a^	303.91^b^	41.2	0. 001	**
**Ig G (mg/dL)**	181.41^b^	182.38^b^	185.73^b^	194.19^a^	201.31^a^	194.27^a^	35.3	0. 001	**
**Total Ig(mg/dL)**	463.3^b^	463.82^b^	470.4^b^	490.2^a^	519.88^a^	498.18^a^	44.6	0.004	**

SEM, standard error of means**. a, b, c…** means in the same Column within each classification bearing different letters are significantly different. sig. =Significance. NS=non-significant. V=Vehicle control (sterilized dimethyl sulfoxide (DMSO).T2 =2mg Grape Pomace dissolved in DMSO, T4=4mg Grape Pomace dissolved in DMSO, T6 =6mg Grape Pomace dissolved in DMSO.

## DISCUSSION

Our findings showed that elevated concentrations of bioactive phenolics, encompassing gallic acid, chlorogenic acid, catechin, syringic acid, resveratrol, hesperetin, and naringenin, in the grape pomace derived from red grape varieties cultivated in Egypt., among others, contains substantial levels of polyphenolics such as resveratrol, catechins, flavonols, and anthocyanins, which exhibit robust antioxidant properties by effectively scavenging free radicals. This antioxidative efficacy can be attributed to the presence of bioactive antioxidants like chlorogenic acid, catechin, resveratrol, hesperetin, and other phenolics within the grape pomace (Ky and Teissedre, 2015; Chedea et al., 2018) .

Eggs injected with the highest concentration of grape pomace extract (**GPE**) at 6 mg per egg exhibited significantly lower hatchability compared to other groups. Conversely, eggs injected with 4mg per egg of GPE showed improved hatchability. It appears that the polyphenolic compounds present in GPE, particularly at the 4 mg level, may aid chicks in overcoming oxidative stress during hatching and exhibit an inhibitory effect on bacteria, as suggested by (Perumalla and Hettiarachchy, 2011). Additionally, the inclusion of nutrients with antioxidant properties, as highlighted by (Surai, 1999), could enhance the antioxidative status and protect developing chicks from oxidative damage. Higher doses of GPE failed to positively influence hatchability, possibly due to alterations in the pH and osmolality of the egg environment or adverse effects of tannins. Conversely, lower levels of GPE (2 mg) were not as effective, likely due to their limited bioavailability, as noted by (Hajati et al., 2014).

Conversely, in ovo injection (**IOI**) has the potential to induce mild stress through the injection process, as noted by (De Smit et al., 2006) and (Everaert et al., 2007). This injection method may pose risks to the internal environment, potentially leading to a detrimental impact on embryo viability and reduced hatchability, as observed by (Daneshyar et al., 2010). However, (Ebrahimi et al., 2012) presented findings suggesting that the in ovo injection of antioxidants during an early stage of embryonic development results in lower hatchability. Furthermore, earlier research has yielded contradictory findings regarding hatchability outcomes. The variations in results can be ascribed to factors like the delivery method, the nature of the solution administered, the type of diluent used, the timing of nutritive solution inoculation, the targeted inoculation area (air cell, amnion, or yolk), and the injection dosage, as highlighted by (de Oliveira et al., 2014) and (Ohta and Kidd, 2001). Each of these factors may have a pivotal role in determining the efficacy of pre-hatching nutrition for chicks.

However, The present results are in contrast to those of (Amina et al., 2019) who documented a higher hatchability percentage of fertile eggs following the injection of the yolk sac with 50 μg of resveratrol (RV/egg) on the 14th d of incubation. This disparity in results may be attributed to the potential enhancement of the antioxidant status of the eggs, assisting the embryo in overcoming oxidative stress during hatching. The enhanced hatchability percentage noted in eggs injected with resveratrol on the 14th d of incubation, a crucial period for fatty acid oxidation, might be attributed to a decrease in the generation of free radicals that can cause substantial damage to cellular membranes. This mechanism could also elevate lipid utilization for energy production, thereby contributing to the observed improvement in hatchability, as suggested by (Schaal, 2008).

Similarly, (Hajati et al., 2014) documented a significant improve in hatchability in broiler breeder hens when injected with 4.5 mg of grape seed extract (GSE/egg) on the 18th d of incubation. Additionally, (Khaligh et al., 2018) Showed that eggs injected with flavonoids into the amnion exhibited greater overall hatchability on the 18th d of embryonic development compared to those not injected.

It has been emphasized that the weight of chicks, at hatching plays a role, in determining their eventual marketing weight (Sklan et al., 2003). Additionally, (Wilson, 1991) Stated that for every gram increase in body weight at hatch, there is an 8 to 13 gram increase in body weight at marketing. However, (Uni et al., 2005) discovered that in ovo feeding with a solution containing maltose, sucrose, dextrin, and HMB6 injected into the amnion on the 17.5th d of incubation resulted in a 5 to 6% rise in hatching weights compared to controls. Moreover, they demonstrated that a 2 gram difference in body weight at hatch due to in ovo feeding led to a 50 to 60 gram increase in body weight by d 25.

Interestingly, the outcomes of the current experiment align with those of (Amina et al., 2019), who demonstrated that chicks' weight at hatch significantly increased (*p* ≤ 0.001) in the group where the yolk sac was injected with 50 μg of resveratrol per egg on the 14th d of incubation, in comparison to the sham or intact control groups. The increase in weight could be ascribed to the enhanced antioxidant status of the embryos. Alleviating oxidative stress associated with hatching might result in higher hatch weights and improved post-hatch performance by protecting skeletal muscle stem cells from oxidative damage, as proposed by (Choi et al., 2016).

Additionally, (Hajati et al., 2014) found that in ovo feeding with a combination of grape seed extract (GSE) and vitamin enhanced performance of hatched chicks. This suggests that grape seed extract could function as a potent anti-stress additive during the incubation time, contributing to the enhancement of broiler performance.

Our findings reveal that grape pomace contains key active ingredients such as Gallic acid and Chlorogenic acid, known for their diverse biological functions comprising properties such as antioxidants, antimicrobials, antivirals, and anti-inflammatory agents (Liang and Kitts, 2015; Lu et al., 2020) Additionally, Gallic acid or its metabolites have been shown to increase the ratio of villus height to crypt depth in broilers as indicator for improving intestinal morphology, thereby enhancing absorption and digestibility (Samuel et al., 2017). Furthermore, Gallic acid demonstrates antibacterial properties against pathogenic through modifications in membrane characteristics. This compound also mitigates oxidative stress and inflammatory responses, while encouraging beneficial alterations in intestinal microbiota and metabolites, potentially supporting both host and intestinal health (Yang et al., 2021). Studies have shown that Chlorogenic acid (**CGA**) governs the chicken's gut microbiota, enhances barrier function, and modulates immune activity (Zha et al., 2023). Enhanced growth performance observed in broiler chickens treated with Chlorogenic acid may be partly ascribed to an improvement in antioxidant capacity(Zha et al., 2023) Elevated nutrient digestibility and heightened activities of digestive enzymes (Chen et al., 2018) Enhanced integrity of the intestine and function of its barrier (Chen et al., 2023) Enhanced immune response (Zhang et al., 2020) Enhanced composition of the gut microbiota (Chen et al., 2021) Reported to play a role in the enhanced growth of poultry, but further investigation is required to determine whether these beneficial effects also explain the improved growth performance observed in our study. Our findings indicate that the improvement in productive performance persists until market age. The enhanced LBW, BWG, and FCR could be attributed to the boosted antioxidant status and immune response at hatch time, which remained consistent until market age. It was noted that (Lu et al., 2007) thyroid hormone levels showed a positive correlation with chick embryonic weight. It was suggested that thyroid hormones might play a critical role in ensuring normal growth and development during chick embryogenesis. According to our findings, it can be deduced that grape pomace extract improved thyroid function by increasing the levels of T3 and T4 in plasma, consequently resulting in enhanced growth compared to the control group. Furthermore, the improved condition of the intestinal tract in chicks injected with in ovo grape pomace extract likely played a role in promoting a favorable gut microbial profile, thus creating conducive conditions for enhanced performance. This finding aligns with previous studies by various authors, including (Abu Hafsa and Ibrahim, 2018; Das et al., 2020; Erinle et al., 2022; Pascual et al., 2022).

Blood serves as a significant medium for assessing the physiological and health status of animals reliably (Erinle et al., 2022). There is scant literature documenting on the effects of in ovo injection of (**GPE**) on the plasma biochemical indices of broiler chickens. Our results reveal that in ovo injection increased plasma TP, ALB, and HDL, and decreased ALT, AST, LDL, and triglycerides. Serum concentrations of TP and ALB are indicative of protein metabolism and nutritional status, serving as the essential components for the synthesis of immunoglobulins (Banales et al., 2019). These findings suggest that GPE may enhance protein synthesis, thereby benefiting the growth and immunity of broilers (Niu et al., 2022) as indicated by an improved immune response in our study through increased immunoglobulin (IgM and IgG) and this is following (Ebrahimzadeh et al., 2018) who found that increased IgG levels with increased levels of Grape Pomace. Also, dietary 0.2% grape pomace extracts enhanced immune response in broilers(Pascual et al., 2022) Polyphenolic compounds obtained from medicinal plants have been noted for their potential to provide immune protection and modulation in poultry organisms (Darmawan et al., 2022), chlorogenic acid (CGA) alleviate hepatic inflammation in broilers challenged with diquat (**DQ**) (Zha et al., 2023) and improved the immunological functions of stressed broilers (Liu et al., 2023a) CGA feeding in broilers exposed to high stock density resulted in downregulated expression of TNF-α, IL-1β, and IL-6 (Liu et al., 2023b). moreover, elevated plasma immunoglobulin (IgG and IgM) levels in Mandara hatched chicks due to in ovo injection of resveratrol (Amina et al., 2019).

Grape pomace has the potential to be a promising natural source of antioxidants, which may have a beneficial effect on cholesterol levels (Dupak et al., 2021). Our results showed that in ovo injection with grape pomace extract has a positive effect on lipid metabolism, especially 4 mg group, this group exhibited the lowest level of cholesterol and triglycerides while the highest level of HDL compared to other levels and the control group. this positive effect of grape pomace extract may be due to its content of anthocyanin (Aditya et al., 2018). This effect can be attributed to polyphenol compounds such as catechin, Gallic acid, and epicatechin. These compounds function by inhibiting the activity of pancreatic cholesterol esterase, an enzyme responsible for breaking down cholesterol esters in diet, thereby releasing free cholesterol. Additionally, these phenolic compounds bind to bile acids, reducing the solubility of cholesterol in micelles and consequently diminishing the absorption of cholesterol (Hajati et al., 2015). our results showed that grape pomace contains a total flavonoid of about (7.42 mg/g), these flavonoids combined with cholesterol in the digestive system, resulting in the formation of new compounds that were subsequently excreted from the intestine without absorption. In this investigation, flavonoids likely played a pivotal role in the reduction of cholesterol levels (Li et al., 2016). Similarly, (Amina et al., 2019) reported a significant (*p* ≤ 0.001) reduction in plasma cholesterol levels in chicks hatched from eggs injected with resveratrol. Additionally, (Feng et al., 2017) observed a decrease in cholesterol status with dietary resveratrol supplementation in a layer diet at concentrations of 0.5, 1.0, 2.0, and 4.0 g/kg. Furthermore, (Abu Hafsa and Ibrahim, 2018) found that broilers fed a diet supplemented with grape seeds at concentrations of 10, 20, and 40 g/kg exhibited reduced levels of plasma total lipids and cholesterol compared to the control birds.

Chick embryo tissues possess a significant concentration of highly polyunsaturated fatty acids in their lipid composition, necessitating the presence of antioxidant defenses. Indeed, the elevated levels of endogenous antioxidants present in the egg and embryonic tissues can function as a significant adaptive mechanism to safeguard the tissue during the oxidative stress encountered at hatching (Madkour et al., 2015; El-Wardany et al., 2016; Surai et al., 2016). The current study's results indicate an enhancement in antioxidant status, manifested by an increase in Total Antioxidant Capacity (**TAC**) and a decrease in Malondialdehyde (MDA) levels in hatched chicks from grape pomace extract (**GPE**) treatments. This improvement may be attributed to the significant quantities of total phenolic content (16.07 mg/g), total flavonoid content (7.42 mg/g), and total anthocyanin (8.37 mg/g) present in GPE in our study. Additionally, GPE's elevated levels of phenolic compounds, such as gallic acid, chlorogenic acid, catechin, syringic acid, resveratrol, hesperetin, and naringenin, contribute to its robust antioxidant effects observed in DPPH scavenging and reducing power assays. These results are in agreement with (Amina et al., 2019) In their study, they observed significant disparities (*p* ≤ 0.0001) in the plasma total antioxidant capacity (**TAC**), superoxide dismutase (**SOD**), and malondialdehyde (**MDA**) activities among hatched chicks after in ovo resveratrol injection. Notably, the groups subjected to in ovo resveratrol injections exhibited substantially higher plasma TAC and SOD levels compared to the intact or sham control groups. Moreover (Abu Hafsa and Ibrahim, 2018) found that the supplementation of 40 g of grape seed resulted in a significant enhancement of antioxidant capacity and a reduction in the lipid peroxidation marker in broilers. (GPE) was identified as having high levels of phenolic and flavonoid compounds, known for their antioxidant properties. This implies that (GPE) has the potential to decrease reactive free radicals, consequently mitigating oxidative damage to tissues, which can positively impact the immunity and growth performance of hatched chicks.

## CONCLUSIONS

The grape pomace extract (**GPE**) is rich in total phenolics, total flavonoids, and total anthocyanins. Additionally, it contains substantial quantities of bioactive phenolics, including gallic acid, chlorogenic acid, catechin, syringic acid, resveratrol, hesperetin, and naringenin. GPE has demonstrated notable antioxidant properties and a robust ability to eliminate free radicals. The in ovo injection of grape pomace extract, especially at a dose of 4 mg, has shown enhancements in growth performance, immune response through increased IgM and IgG, and antioxidant status through increased total antioxidant capacity and reduced MDA in hatched chicks. This positive impact has persisted until the market age. So administering natural antioxidants (such as grape pomace extract) to developing broiler embryos via in ovo feeding into the amnion could serve as a valuable strategy for enhancing the subsequent post-hatch productive performance, as well as bolstering the antioxidant and immunological status of broiler chicks.

## DISCLOSURES

The authors declare no conflicts of interest.
